# Age-Related Changes in Stand Structure, Spatial Patterns, and Soil Physicochemical Properties in *Michelia macclurei* Plantations of South China

**DOI:** 10.3390/life15060917

**Published:** 2025-06-05

**Authors:** Jiaman Yang, Jianbo Fang, Dehao Lu, Cheng Li, Xiaomai Shuai, Fenglin Zheng, Honyue Chen

**Affiliations:** 1 College of Forestry and Landscape Architecture, South China Agricultural University, Guangzhou 510642, China; 17819564956@163.com (J.Y.); qw875363261@163.com (D.L.); licheng@stu.scau.edu.cn (C.L.); 17607331801@163.com (X.S.); zhengfenglin-1@163.com (F.Z.); 2 Eco-Environmental Monitoring and Research Center, Pearl River Valley and South China Sea Ecology and Environment Administration, Ministry of Ecology and Environment of the People’s Republic of China, Guangzhou 510611, China; fjianbo@outlook.com

**Keywords:** *Michelia macclurei* plantation, stand age, stand structure, soil physicochemical properties

## Abstract

*Michelia macclurei*, a valuable native broad-leaved species with good ecological and economic benefits and a key afforestation tree in South China, is facing progressive stand degradation and soil fertility decline with age. To investigate age-dependent dynamics of stand structure and soil properties, this study examined five stands (5, 10, 15, 20, and 42 a) in Yunfu City, Guangdong Province. The results revealed that (1) spatial distribution shifted from aggregated in young stands (5–10 a) to random in mature stands (42 a), with diameter and height class distributions becoming more diverse with age. Notably, topsoil (0–20 cm) in near-mature stands (15–20 a) exhibited not only significantly higher capillary porosity, non-capillary porosity, and water-holding capacity compared to young stands but also increased bulk density, indicating soil physical degradation. (2) Soil nutrient decline was observed in over-mature stands (42 a), characterized by a reduction in soil total nitrogen to 1.08 ± 0.06 g·kg^−1^ and total phosphorus to 0.16 ± 0.02 g·kg^−1^ in the topsoil (0–20 cm layer), suggesting age-related soil nutrient degradation. (3) Correlation analysis revealed a significant negative correlation between total potassium content and crown uniformity indices (*p* < 0.01), while available phosphorus was significantly positively correlated with crown and tree growth (*p* < 0.05). These findings provide critical insights for developing stage-specific management strategies in *Michelia macclurei* plantations.

## 1. Introduction

Plantation forests play an irreplaceable role in the reserve of forest resources and the restoration of ecosystems [1]. However, monoculture plantations, due to their homogeneous stand structure, often face challenges as they mature—most notably soil fertility decline and reduced productivity [2]. Although numerous studies confirm these trends, controversy persists regarding the primary drivers: some research highlights the influence of stand structure on the spatial distribution of soil nutrients—where “soil nutrient dynamics” (i.e., the spatial and temporal distribution and fluctuations of soil nutrients) is modulated by the arrangement of trees [3,4,5]—while other studies suggest that rapid tree growth leads to cumulative nutrient depletion [6,7]. Moreover, the spatial configuration of the stand governs these nutrient dynamics. Likewise, indices such as the angular distribution index—which quantifies tree orientation and arrangement to influence microclimatic conditions and soil and water conservation capacity—along with canopy openness and stratification index, play critical roles in modulating the soil’s physicochemical properties [8]. Previous studies indicate that as plantation forests mature, marked depletion in soil nutrient reserves, including organic carbon, results in significant soil fertility degradation and a reduction in productivity by up to 20% [6]. The stand architecture across varying age classes induces distinct feedback mechanisms on soil nutrient availability.

In the context of subtropical forestry, *Michelia macclurei*, an evergreen broad-leaved tree species within the Magnoliaceae family and *Michelia* genus, is a significant indigenous tree species in southern China [9]. Renowned for its fast-growing nature, stress tolerance, and high-quality timber properties, as well as its ecological service functions such as carbon sequestration, oxygen release, soil and water conservation, and fire and wind resistance [10]. Its widespread application in urban greening, ecological restoration, and large-diameter timber cultivation [11,12,13] underscores its importance as a model species for sustainable forest management. However, prolonged artificial management in monoculture plantations has resulted in issues, including soil fertility degradation and reduced biodiversity [14,15,16]. Although previous research on *Michelia macclurei* plantations has predominantly focused on physiological characteristics such as wood formation [17,18] and photosynthesis [19]. Long-term studies on the evolution of stand structure and soil properties, especially in mature stands (more than 40 years old), are still lacking. This gap is especially significant within the broader research on subtropical plantation forestry, where integrated studies on long-term stand development and soil evolution are rare.

In this context, our study examines *Michelia macclurei* plantations across five stand ages (5, 10, 15, 20, and 42 a), focusing on whether stand structure exhibits stage-specific differentiation with increasing stand age and whether soil degradation occurs at specific stages. This study aimed to elucidate the mechanisms by which stand structure influences soil nutrient dynamics during critical developmental stages, thereby providing a theoretical basis for sustainable management practices such as soil fertility maintenance and stand density regulation.

## 2. Materials and Methods

### 2.1. Study Area

The study area is located in the Yunfu Forest Farm in Guangdong Province (111°03′–112°31′ E and 22°22′–23°19′ N), characterized by a subtropical monsoon climate. The terrain is higher in the southwest and lower in the northeast, falling within a low mountainous and hilly region, with elevations ranging between 100 and 600 m above sea level. The region experiences an annual average temperature of 20–22 °C and receives annual precipitation ranging from 1300 to 1800 mm. The predominant soil in the study area is a lateritic red soil with a loamy clay texture, which is classified under the World Reference Base for Soil Resources (WRB) as Ferralsol.

The investigated stands consist of pure *Michelia macclurei* plantations aged 5, 10, 15, 20, and 42 a. The dominant tree species in the study area include *Castanopsis hystrix*, *Manglietia conifera*, *Michelia odora*, *Cunninghamia lanceolata*, *Eucalyptus*, and *Pinus massoniana*. The understory shrub layer is primarily composed of species such as *Persicaria chinensis*, *Mussaenda pubescens*, *Machilus chinensis*, and *Psychotria rubra*. The herbaceous layer is dominated by *Pteris semipinnata*, *Blechnopsis orientalis*, *Dicranopteris pedata*, and *Melicope pteleifolia*.

### 2.2. Experimental Design

In April 2024, pure *Michelia macclurei* plantations with consistent site conditions were selected as this study’s objects within an elevation range of 100–350 m in the Yunfu Forest Farm, Guangdong Province. For each of the five stand ages (5, 10, 15, 20, and 42 a), three 20 m × 20 m plots were established, totaling 15 plots (Table 1). All plots were located on shaded slopes, with similar slope aspects and gradients across replicates. Sample plots of different age groups should be spaced at least 50 m apart. *Michelia macclurei* trees of different age classes receive the same management measures—such as fertilization, pruning, and other maintenance—only during the initial planting period (within three years); no management is carried out thereafter.

The surveyed stand factors included diameter at breast height (DBH), tree height, height under the crown, and crown width. And we adopted the formula from previous research, V = 0.667054 × 10^−4^ × D^1.847954450^ × H^0.96657509^, to calculate the individual tree volume of *Michelia macclurei*. Trees with a DBH greater than 5 cm within the plots were georeferenced and numbered using a Real-Time Kinematic (RTK) system (Qianxun Star SR6, Hangzhou, China). Litter and humus were removed from the sampling points. Along the diagonal of each plot, three sampling points (upper, middle, and lower positions) were established. Soil samples were collected using a soil core sampler at three depth intervals: 0–20 cm, 20–40 cm, and 40–60 cm. Three soil core samples were taken from each depth interval for the determination of soil physical properties. Additionally, approximately 500 g of soil was collected from each sampling point at the corresponding depth, thoroughly mixed, and transported to the laboratory. After air-drying and sieving, the samples were used for analyzing soil chemical properties.

### 2.3. Stand Diameter Class and Height Class Structure

The methods for calculating the diameter class and height class structure of the stands in this study are as follows [20]: DBH was divided into 2 cm intervals for each diameter class, and tree height was grouped into 5 m intervals for each height class. The DBH and height sequences of trees within each sample area were classified into these intervals, and the frequency of trees in each class was counted and converted into a percentage. This provided the frequency distribution of diameter and height classes within the stands, which was then visualized using histograms. In this study, the calculation methods for stand diameter class and height class structures followed those of the previous publication [20]. In order to obtain information such as stand closure and leaf area index, three points were randomly selected in each sample plot (with a spacing of more than 5 m). Using a Canon camera (Canon EOS 50D Mark II, Canon Inc., Tokyo, Japan) equipped with a fisheye lens (Canon EF 8–15mm f/4L USM, Canon Inc., Tokyo, Japan), vertical photographs of the canopy were taken from a height of 50 cm above the ground under clear weather conditions, and the collected canopy images were subsequently analyzed using Gap Light Analyzer (GLA, Version 2.0).

### 2.4. Crown Structure

Crown structure is one of the key indicators reflecting the long-term growth status, competitive dynamics, and health condition of trees [21]. It plays a decisive role in determining tree productivity and the full realization of ecological benefits [22]. In this study, parameters such as crown width (*CW*), crown length (*CL*), and live crown ratio (*CR*) were used to describe the size of the crown in horizontal or vertical dimensions. Meanwhile, crown projection ratio (*CPR*), crown surface area (*CSA*), and crown volume (*CV*) were employed to characterize the three-dimensional spatial attributes of the crown. The formulas for calculating these parameters are as follows:

(1) $$CW=(CW_{ew}+CW_{sn})/2$$

(2) CR=CL/H

(3) CL=H−HCB

(4) CPR=CW/DBH

(5) $$CSA=\pi \times CW/4\times \sqrt{{CL}^{2}+{CW}^{2}/4}$$

(6) $$CV=\pi \times CW_{ew}\times CW_{sn}\times \frac{CL}{12}$$

In this formulas, *CW_ew_* and *CW_sn_* represent the east-west crown width and the north-south crown width, respectively; *H* denotes tree height; *DBH* is the diameter at breast height; and *HCB* is the height under the crown.

### 2.5. Stand Spatial Distribution Pattern

The spatial structure of a stand determines the competitive relationships and positional arrangements among trees, playing a critical role in forest management and stand stability [23]. Since this study focuses on pure *Michelia macclurei* plantations with no species mixing, only two spatial structure indices (uniform angle index and neighborhood comparison) were selected to analyze the horizontal distribution pattern and competition intensity among trees.

The uniform angle index is a key parameter for describing the uniformity of neighboring trees around a reference tree, providing an accurate assessment of the horizontal spatial distribution pattern of trees [24]. The calculation formulas are as follows (Equations (7) and (8)):

(7) $$W_{i}=\frac{1}{4}\sum_{j=1}^{4}Z_{ij}$$

(8) $$\bar{W}=\frac{1}{N}\sum_{j=1}^{N}W_{i}$$

In these formulas, *W_i_* represents the uniform angle index for the reference tree *i*, and *Z_ij_* is a discrete variable defined such that *Z_ij_* = 1 when the standard angle *α*_0_ is greater than the *j*-th angle *α*; otherwise *Z_i_* = 0. $\bar{W}$ denotes the mean uniform angle index for the stand.

The neighborhood comparison is a key parameter for describing the degree of size differentiation among trees [25]. The calculation formulas are as follows (Equations (9) and (10)):

(9) $$U_{i}=\frac{1}{n}\sum_{j=1}^{n}K_{ij}$$

(10) $$\bar{U}=\frac{1}{N}\sum_{j=1}^{n}U_{i}$$

In these formulas, *U_i_* represents the neighborhood comparison for the reference tree *i*; *n* is the number of neighboring trees; *K_ij_* is a discrete variable, where *K_ij_* = 1 if the *DBH* of the reference tree *i* is smaller than that of the neighboring tree *j*; otherwise *K_ij_* = 0; and $\bar{U}$ denotes the mean neighborhood comparison for the stand.

### 2.6. Determination of Soil Physicochemical Properties

In accordance with the previously reported methods [26], soil moisture content was measured using the oven-drying method, while soil bulk density, total porosity, capillary porosity, and soil water-holding capacity were determined using the soil core method. The saturated water-holding capacity (*W_t_*, t·hm^−2^), capillary water-holding capacity (*Wa*, t·hm^−2^), and non-capillary water-holding capacity (*W_b_*, t·hm^−2^) were selected as key indicators for evaluating soil water conservation capacity [27]. The calculation formulas are as follows:

(11) $$W_{t}=10000\times P_{t}\times h$$

(12) $$W_{a}=10000\times P_{a}\times h$$

(13) $$W_{b}=10000\times P_{b}\times h$$

In these formulas, *P_t_* represents the total soil porosity (%), *P_a_* represents the capillary soil porosity (%), *P_b_* represents the non-capillary soil porosity (%), and *h* represents the soil layer thickness (m).

Soil organic carbon content was determined using the potassium dichromate volumetric method-external heating method. Soil pH was measured using the potentiometric method (soil-to-water ratio of 2.5:1). Total nitrogen content was determined using the Kjeldahl method. Total phosphorus content was measured using the sodium hydroxide fusion-molybdenum antimony colorimetric method. Total potassium content was determined using the sodium hydroxide fusion-flame photometry method. Alkali-hydrolyzable nitrogen content was measured using the alkali diffusion method. Available phosphorus content was determined using the molybdenum antimony colorimetric method. Available potassium content was measured using flame photometry. Detailed experimental methods refer to the national standard of China: GB 5009.15-2014 [4,26].

### 2.7. Data Processing and Statistical Analysis

Data were processed using Microsoft Excel 2019. The data underwent normality and homogeneity of variance tests before processing, and the results indicated that it follows a normal distribution. Stand spatial structure analysis was conducted using the Winkelmass 1.0 software. Distribution histograms and curves for diameter class structure, height class structure, uniform angle index, and neighborhood comparison were plotted using Origin 2024. One-way analysis of variance (ANOVA) and Duncan’s multiple range test were performed using SPSS 22.0 to analyze stand growth indicators, crown structure, spatial structure indices, and soil physicochemical properties. Statistical significance was assessed at the *p* < 0.05 level. Additionally, for the correlation analysis of structure and soil physical and chemical properties of *Michelia macclurei* plantations of different ages, models of the form *Plant_variable* ~ *Soil_variable* + (1|*Stand*) (where the random effect (1|*Stand*) corrects for spatial dependencies within nested plots) were fitted. The marginal R^2^ (i.e., the variance explained by the fixed effect) was extracted using the MuMIn package in R 4.4.1 and then converted into a correlation coefficient using the equation *R = sign*(*β*) × √(*R*^2^), with *β* representing the regression estimate for the soil variable.

## 3. Results

### 3.1. Stand Growth of Michelia macclurei Plantations Across Different Stand Ages

The tree height, DBH, and crown width of the 42-year-old stand increased significantly by 2.39 times, 1.41 times, and 1.67 times, respectively, compared to the 5-year-old stand (Table 2). This indicates that as stand age increases, the tree height, DBH, and individual tree volume gradually increase. In contrast, the crown width exhibited the following trend across stand ages: 42 a (7.58 m) > 20 a (5.29 m) > 10 a (5.01 m) > 15 a (3.63 m) > 5 a (2.84 m) (Table 2).

### 3.2. Diameter Class and Height Class Structures of Michelia macclurei Plantations with Different Stand Ages

**Figure 1:**
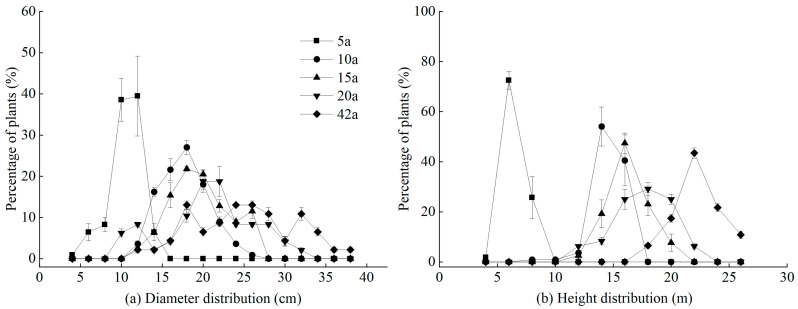
Diameter structure (**a**) and height structure (**b**) of *Michelia macclurei* plantations at different forest ages.

The diameter class structure of *Michelia macclurei* plantations exhibited significant differences across different stand ages, all displaying a normal distribution (Figure 1a). As stand age increased, the number of diameter classes increased, and the peak of the diameter class distribution gradually shifted to larger values (Figure 1a). In the 5-year-old stand, the diameter class frequency distribution was left-skewed, with the majority of trees concentrated in the 10–12 cm range, peaking at the 12 cm diameter class and reaching the lowest frequency at the 4 cm diameter class (Figure 1a). In the 10-year-old stand, the diameter class frequency distribution was also left-skewed, with trees primarily concentrated in the 14–22 cm range, peaking at the 18 cm diameter class and reaching the lowest frequency at the 26 cm diameter class (Figure 1a). In the 15-year-old stand, the diameter class frequency distribution followed a normal distribution, with trees mainly concentrated in the 16–26 cm range, peaking at the 18 cm diameter class and reaching the lowest frequency at the 12 cm diameter class (Figure 1a). In the 20-year-old stand, the diameter class frequency distribution was normal, with trees concentrated in the 18–28 cm range (Figure 1a). In the 42-year-old stand, the diameter class frequency distribution was right-skewed, with trees concentrated in the 18–32 cm range (Figure 1a).

The height class structure of *Michelia macclurei* plantations varied significantly across different stand ages, all exhibiting a unimodal pattern that approximates a normal distribution (Figure 1b). As the height class gradient increased, the number of trees initially rose and then declined. In the 5-year-old stand, the tree height distribution was primarily concentrated in the 6–8 m range, accounting for 98.16% of the total trees (Figure 1b). In the 10-year-old stand, the tree height distribution was mainly concentrated in the 14–16 m range, representing 94.59% of the total trees (Figure 1b). In the 15-year-old and 20-year-old stands, the tree height distribution was concentrated in the 14–20 m range, accounting for 97.43% and 87.5% of the total trees, respectively (Figure 1b). In the 42-year-old stand, the tree height distribution was concentrated in the 20–26 m range, representing 93.49% of the total trees (Figure 1b).

### 3.3. Crown Structure and Spatial Structure of Michelia macclurei Plantations Across Different Stand Ages

The crown structure indicators of *Michelia macclurei* plantations exhibited significant differences across different stand ages (Table 3). The crown volume and crown surface area of the 42-year-old stand were 7.77 times and 22.61 times greater, respectively, than those of the 5-year-old stand (Table 3). The live crown ratio of the 15-year-old stand was lower than that of the other four stand ages (Table 3). The crown projection ratio showed a trend of initially decreasing and then increasing with stand age, with values ranked as follows: 42 a (0.31) > 5 a (0.28) > 20 a (0.26) > 15 a (0.24) > 10 a (0.23) (Table 3).

**Figure 2:**
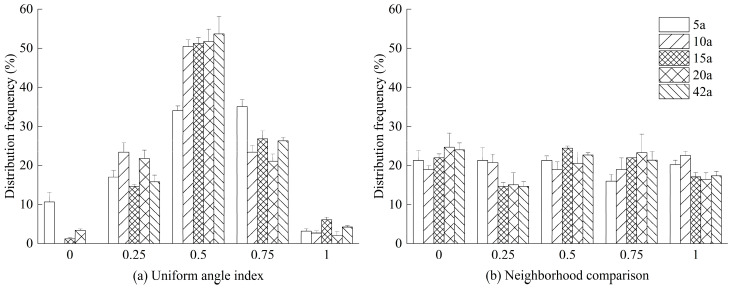
Distribution frequencies of uniform angle index (**a**) and neighborhood comparison (**b**) of *Michelia macclurei* plantations at different forest ages.

From the frequency distribution of five spatial distribution states (*W_i_* = 0, 0.25, 0.5, 0.75, and 1) across different stand ages of *Michelia macclurei* plantations, it is evident that the probabilities of *W_i_* = 0 and *W_i_* = 1 were both below 10% (Figure 2), indicating that structural units exhibiting absolute regularity or absolute irregularity were rare across these stand ages. Among all distribution states, the probability of the uniform angle index being 0.5 was the highest (34.05–53.68%) for all five stand ages, suggesting that the majority of structural units were in a random distribution state (Figure 2a). The univariate distribution characteristics of the uniform angle index across different stand ages generally followed a normal distribution, while the frequency distribution of the stand mean diameter neighborhood comparison was relatively balanced across all classes, each approaching approximately 20% (Figure 2).

The uniform angle index of *Michelia macclurei* plantations varied significantly across different stand ages (Table 4). Specifically, the uniform angle index of the 5-year-old stand (0.5720) was significantly higher than that of the 20-year-old stand (0.4883), while the uniform angle indices of the 10-year-old, 15-year-old, and 42-year-old stands showed no significant differences compared to the other two stand ages (*p* ≥ 0.05) (Table 4). The uniform angle index of the 5-year-old stand was 0.5720, and that of the 20-year-old stand was 0.4883 (Table 4), indicating that the overall distribution pattern of these stands exhibited random distribution characteristics. The uniform angle indices of the 10-year-old and 15-year-old stands ranged between 0.51 and 0.55 (Table 4), suggesting a clustered distribution pattern. In contrast, the diameter neighborhood comparison showed no significant differences across stand ages (Table 4), indicating that the structural units were uniformly distributed. The neighborhood comparisons were ranked as follows: 10 a (0.5137) > 15 a (0.4936) > 42 a (0.4902) > 5 a (0.4854) > 20 a (0.4795). The mean diameter neighborhood comparison of the stands was 0.4925 (Table 4), indicating that the trees exhibited no significant dominance or suppression, and the stands were in an intermediate state.

### 3.4. Soil Physical Properties of Michelia macclurei Plantations Across Different Stand Ages

The soil physical properties of *Michelia macclurei* plantations varied significantly across different stand ages (Table 5). As soil depth increased, the soil bulk density of *Michelia macclurei* plantations gradually increased, with significant differences observed among stand ages (Table 5). The topsoil bulk density of the 42-year-old stand (1.10 g·cm^−3^) was significantly higher than that of other stand ages, while the 5-year-old stand had the lowest topsoil bulk density (0.99 g·cm^−3^) (Table 5). In contrast, the deeper soil layers (40–60 cm) exhibited higher bulk density across all stand ages, ranging from 1.26 to 1.40 g·cm^−3^ (Table 5). Soil total porosity and saturated water-holding capacity significantly decreased with increasing soil depth (Table 5). The topsoil layer (0–20 cm) of near-mature stands had significantly higher capillary porosity, non-capillary porosity, capillary water-holding capacity, and non-capillary water-holding capacity compared to young stands (Table 5). However, soil porosity in deeper soil layers was less influenced by stand age (Table 5).

### 3.5. Soil Chemical Properties of Michelia macclurei Plantations Across Different Stand Ages

The soil nutrient content of *Michelia macclurei* plantations varied significantly across different stand ages within the same soil layer (Table 6). The soil pH of *Michelia macclurei* plantations ranged from 4.15 to 4.51 (Table 6), indicating weakly acidic conditions overall. The deep soil layer of the 42-year-old stand had the highest pH value (4.51), while the topsoil layer of the 10-year-old stand had the lowest pH value (4.15) (Table 6). With increasing stand age, the average soil organic matter, total nitrogen, and total phosphorus content initially increased and then decreased, while the average total potassium content showed an overall increasing trend (Table 6). The topsoil layer of the 20-year-old stand had the highest total potassium content (17.43 g·kg^−1^), significantly higher than that of other stand ages, while the deep soil layer of the 42-year-old stand reached the highest total potassium content (19.61 g·kg^−1^) (Table 6). The average alkali-hydrolyzable nitrogen and available potassium content initially decreased and then increased with stand age, showing significant differences, whereas the available phosphorus content did not exhibit a clear trend with stand age (Table 6).

As soil depth increased, soil pH gradually increased, showing the trend of 0–20 cm < 20–40 cm < 40–60 cm, and the total potassium content exhibited the same vertical distribution pattern (Table 6). However, the overall soil nutrient content significantly decreased with increasing soil depth, following the trend of 0–20 cm > 20–40 cm > 40–60 cm (Table 6), indicating a pronounced surface accumulation effect. Significant differences were observed between soil layers (Table 6). Among the nutrients, organic matter and total nitrogen showed the largest decreases, while available phosphorus exhibited a relatively smaller decline (Table 6).

### 3.6. Relationship Between Stand Structure and Soil Physicochemical Properties

**Figure 3:**
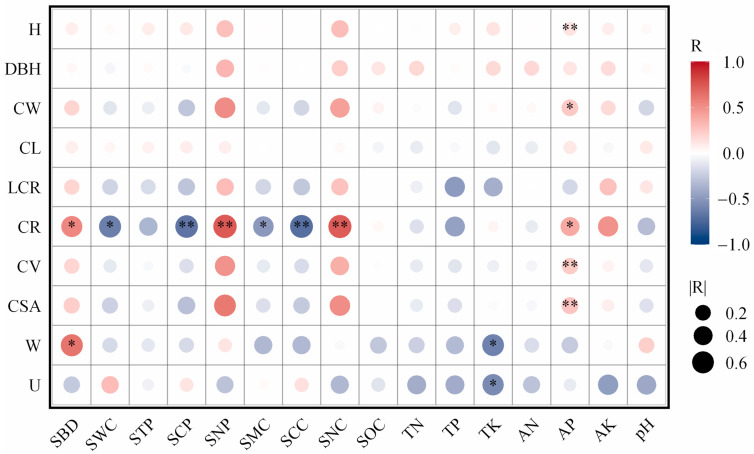
Correlation analysis of structure and soil physical and chemical properties of *Michelia macclurei* plantations of different ages. Note: soil bulk density: *SBD*; soil water content: *SWC*; soil total porosity: *STP*; soil capillary porosity: *SCP*; soil non-capillary porosity: *SNP*; saturated water-holding capacity: *SMC*; capillary water-holding capacity: *SCC*; non-capillary water-holding capacity: *SNC*; soil organic carbon content: *SOC*; total nitrogen: *TN*; total phosphorus: *TP*; total potassium: *TK*; alkali-hydrolyzable nitrogen: AN; available phosphorus: AP; available potassium: *AK*; soil pH: *pH*; crown width: *CW*; crown length: *CL*; live crown ratio: *LCR*; crown projection ratio: *CR*; crown volume: *CV*; crown surface area: *CSA*; uniform angle index: *W*; neighborhood comparison: *U*; tree height: *H*; diameter at breast height: *DBH*; *: *p* < 0.05; **: *p* < 0.01.

Correlation analysis revealed that crown width (CW), crown projection ratio (CR), crown volume (CV), and crown surface area (CSA) of the stands were significantly correlated with several soil physical properties (*p* < 0.05) (Figure 3). Specifically, the crown projection ratio showed highly significant negative correlations with soil water content, capillary porosity, saturated water-holding capacity, and capillary water-holding capacity (*p* < 0.01), while it exhibited highly significant positive correlations with soil non-capillary porosity, non-capillary water-holding capacity, and soil bulk density (*p* < 0.01). The total potassium content was significantly negatively correlated with the uniform angle index and neighborhood comparison (*p* < 0.01) (Figure 3). Additionally, available phosphorus content (AP) was significantly positively correlated with crown width, crown projection ratio, crown volume, crown surface area, and tree height (H).

## 4. Discussion

### 4.1. Age-Dependent Characteristics of Stand Structure

The stand structure of *Michelia macclurei* plantations exhibited significant differences across stand ages, as seen through diameter class, height class, and crown structure variations. During the young stand stage (5–10 years), rapid growth is reflected by a left-skewed diameter distribution, with most trees concentrated in the 10–12 cm range, and a clustered spatial pattern (uniform angle index = 0.5720) that indicates high intraspecific competition. In the middle-aged stage (15–20 years), development becomes stable; the observed reduction in crown width at 15 years may signal a transitional phase in crown development where trees prioritize vertical growth to capture light amid increased competition before lateral expansion resumes. In the mature stand stage (42 years), pronounced natural thinning is evident, with a diversified diameter class range (18–32 cm) exhibiting a right-skewed distribution and a shift to a random spatial pattern (uniform angle index = 0.4883). The uniform angle index and neighborhood comparison reflect the horizontal distribution pattern and competitive pressure of trees [28]. Although these dynamics are generally consistent with those found in subtropical plantations (e.g., *Cunninghamia lanceolata* [29] and *Sonneratia apetala* [30]), the transition from a clustered to a random distribution in *M. macclurei* occurs at 15–20 years—a delay of approximately 5–10 years compared with *Larix principis* [31]—possibly due to its slower early growth rate. Likewise, comparisons with *Cunninghamia lanceolata* highlight species-specific crown development and resource allocation strategies that influence stand heterogeneity. Therefore, in the young stand stage, it is recommended to remove diseased, weak, and overcrowded trees during thinning and adjust spacing to promote individual growth, and consider introducing native species such as *Cunninghamia lanceolata* for mixed-species transformation to improve stand spatial heterogeneity and reduce competition. Empirical studies in local agroforestry systems have demonstrated that interplanting with species like *Cunninghamia lanceolata* effectively reduces intraspecific competition and niche overlap, thereby providing a sound basis for recommending mixed-species transformations in *M. macclurei* plantations [32]. In the middle-aged stage, thinning should aim to remove trees with overlapping crowns to maintain good growing space [33,34]. Moreover, similar studies have also shown that plantation trees grow rapidly in the young and middle-aged stages but gradually decline after near-maturity [35]. Therefore, for mature stands, targeted thinning that removes senescent and suppressed trees—thereby opening the canopy and enhancing resource distribution—is pivotal for counteracting growth senescence, boosting natural regeneration, and extending the productive lifespan of *Michelia macclurei* plantations.

### 4.2. Age-Related Characteristics of Soil Degradation

Soil physicochemical properties are critical indicators of plantation ecosystem functions and are closely related to stand growth and soil microbial activity [36]. The age-dependent characteristics of soil physical properties and nutrient content jointly reveal key stages of soil degradation [37,38]. Our study showed that the soil bulk density of 15–20-year-old stands was significantly higher than that of young stands (5 years), while non-capillary porosity (i.e., the fraction of larger soil pores that do not retain water via capillary forces, thereby facilitating drainage and aeration), saturated water-holding capacity, and capillary water retention were significantly lower than in near-mature stands (42 years). These results, along with direct measurements, indicate severe soil compaction likely due to rapid tree growth, extensive root expansion, and litter accumulation [39]. Dense root networks further exacerbate soil compaction, particularly in deeper soil layers, resulting in increased soil bulk density and reduced non-capillary porosity, while litter accumulation alters the pore structure of topsoil. Nutrient analyses revealed that, as stand age increased, topsoil fertility indicators declined: organic matter and total nitrogen content peaked at 15 years before decreasing, whereas total potassium content continuously increased. The 42-year-old stand exhibited a distinct “potassium enrichment-nitrogen and phosphorus depletion” phenomenon, in contrast to the synchronous decline of nitrogen and phosphorus observed in *Tectona grandis* plantations [40]. This difference suggests a nutrient imbalance that may affect long-term soil fertility, thereby emphasizing the need for species-specific nutrient management [41]. Although direct physiological studies on *M. macclurei* are limited, these nutrient trends likely result from a strong potassium uptake capacity combined with a relatively weak nutrient return via litter decomposition. Therefore, during the middle-aged stage, appropriate application of nitrogen and phosphorus fertilizers is recommended to enhance topsoil fertility, while potassium sulfate application in the near-mature stage may prevent excessive potassium enrichment [42,43,44].

### 4.3. Relationship Between Stand Structure and Soil Properties

A well-structured stand and fertile soil are crucial for maintaining the functions of plantation ecosystems [45,46]. Crown metrics—including width, projection ratio, volume, and surface area—were significantly correlated with various soil parameters. For instance, the crown projection ratio displayed highly significant negative correlations with soil water content, capillary porosity, saturated water-holding capacity, and capillary water-holding capacity, suggesting that in soils with high moisture retention and fine pore structures, trees may restrict lateral crown expansion to minimize water loss or optimize resource allocation. Additionally, enhanced litter deposition and increased root exudate inputs promote macropore formation in the topsoil, which improves water infiltration and nutrient movement [47,48]. Moreover, taller trees tend to absorb potassium more rapidly, reducing its availability in the soil, a finding that aligns with the observed negative relationship between total potassium content and crown uniformity parameters [49]. In combination with the significant positive associations between available phosphorus and various crown attributes, these results underscore the critical role of soil structure and nutrient dynamics in influencing forest stand dynamics and tree growth strategies. Consequently, forest management practices should aim to balance soil potassium supply and demand through targeted deep potassium fertilization and stand structure optimization [3,50], while appropriate thinning and additional silvicultural measures during the maintenance phase of near-mature stands can further help reduce stand density and promote natural nutrient cycling [44].

## 5. Conclusions

The stand structure and soil properties in *Michelia macclurei* plantations vary systematically with age, revealing a coherent, age-dependent pattern. Specifically, young stands exhibit a clustered spatial distribution, whereas mature stands transition to a random pattern with increased structural complexity. Moreover, this study uncovered stage-specific soil degradation patterns: a critical phase of soil physical property degradation occurs in 15–20-year-old stands, and in 42-year-old stands, a striking imbalance emerges—marked by potassium enrichment coupled with nitrogen and phosphorus depletion. These patterns not only confirm our initial expectations but also offer novel insights into how the coupling of vegetation structure and soil attributes may differ from other species studied in similar systems, thereby advancing our understanding of plantation dynamics in subtropical environments.

Based on these insights, our management recommendations can be tailored to each developmental stage. For young stands, density regulation and the introduction of mixed species are essential to foster biodiversity and prevent early overcrowding. In the middle-aged phase, targeted interventions such as deep soil loosening combined with nitrogen and phosphorus supplementation are advised to mitigate soil degradation during this vulnerable period. In mature stands, site-specific strategies to regulate potassium levels are necessary to restore nutrient balance and ensure long-term productivity. Recognizing this study’s limitation of examining only five stand ages, future research should implement long-term, experimental studies that incorporate diverse management regimes, climate variability, microbial interactions, and belowground processes. Long-term, experimental studies incorporating different management regimes and mixed-species plantations are essential to validate the patterns observed here and to develop adaptive, evidence-based management practices.

## Figures and Tables

**Table 1:** Basic situation of research sample plots of *Michelia macclurei* plantations of different forest ages.

Stand Age (a)	Altitude (m)	Slope (°)	Slope Aspect	Canopy Density	Stand Density (Plant·hm^−2^)	Understory Vegetation Shannon–Wiener Index
5	322	28	Southeast	0.89	900.00 ± 72.17	2.9631
10	122	22	Southeast	0.92	925.00 ± 28.87	1.7463
15	151	31	Southwest	0.93	650.00 ± 62.92	1.8930
20	243	30	Southwest	0.89	400.00 ± 62.92	2.1030
42	178	36	Southeast	0.88	383.33 ± 46.40	1.9039

Note: All the data in the table are the average values of 3 repeated plots for each stand age.

**Table 2:** The main growth indexes of *Michelia macclurei* plantations at different forest ages.

Stand Age (a)	Tree Height (m)	DBH (cm)	Crown Width (m)	Individual Tree Volume (m^3^)
5	6.48 ± 0.16 d	10.60 ± 0.43 d	2.84 ± 0.23 c	0.03 ± 0.00 d
10	14.63 ± 0.45 c	17.96 ± 0.29 c	5.01 ± 0.21 c	0.19 ± 0.01 c
15	16.30 ± 0.67 bc	19.85 ± 0.21 bc	3.63 ± 0.09 b	0.25 ± 0.01 bc
20	17.97 ± 0.74 b	21.51 ± 1.16 b	5.29 ± 0.78 b	0.32 ± 0.04 b
42	21.96 ± 0.49 a	25.52 ± 1.47 a	7.58 ± 0.33 a	0.53 ± 0.06 a

Note: The data indicate mean ± standard error. Different lowercase letters indicated significant differences at the *p* < 0.05 level.

**Table 3:** Canopy structure of *Michelia macclurei* plantations at different forest ages.

Stand Age (a)	Crown Width (m)	Crown Length (m)	Live Crown Ratio	Crown Projection Ratio	Crown Volume (m^3^)	Crown Surface Area (m^2^)
5	2.84 ± 0.23 c	4.42 ± 0.08 d	0.68 ± 0.02 a	0.28 ± 0.01 ab	10.46 ± 1.06 d	9.74 ± 1.72 c
10	4.01 ± 0.21 bc	9.51 ± 0.10 c	0.65 ± 0.03 a	0.23 ± 0.02 b	31.02 ± 1.48 c	42.33 ± 3.53 c
15	4.63 ± 0.09 b	9.91 ± 0.96 bc	0.60 ± 0.04 a	0.24 ± 0.00 b	38.53 ± 3.49 bc	61.70 ± 6.60 bc
20	5.29 ± 0.78 b	11.38 ± 0.48 b	0.65 ± 0.02 a	0.26 ± 0.04 ab	53.82 ± 11.37 b	111.67 ± 39.47 b
42	7.54 ± 0.33 a	14.84 ± 0.54 a	0.67 ± 0.04 a	0.31 ± 0.01 a	91.76 ± 3.24 a	229.98 ± 13.55 a

Note: The data indicate mean ± standard error. Different lowercase letters indicated significant differences at the *p* < 0.05 level.

**Table 4:** Differences in spatial structure index of *Michelia macclurei* plantations at different forest ages.

Stand Age (a)	Uniform Angle Index	Neighborhood Comparison
5	0.5720 ± 0.0280 a	0.4854 ± 0.0154 a
10	0.5143 ± 0.0135 ab	0.5137 ± 0.0106 a
15	0.5552 ± 0.0187 ab	0.4936 ± 0.0110 a
20	0.4883 ± 0.0120 b	0.4795 ± 0.0060 a
42	0.5010 ± 0.0305 ab	0.4902 ± 0.0151 a

Note: The data indicate mean ± standard error. Different lowercase letters indicated significant differences at the *p* < 0.05 level.

**Table 5:** Soil physical properties of *Michelia macclurei* plantations at different forest ages.

Stand Age (a)	Layer (cm)	Soil Bulk Density (g·cm^−3^)	Soil Total Porosity (%)	Capillary Porosity (%)	Non-Capillary Porosity (%)	Saturated Water-Holding Capacity (t·hm^−2^)	Capillary Water-Holding Capacity (t·hm^−2^)	Non-Capillary Water-Holding Capacity (t·hm^−2^)
5	0–20	0.99 ± 0.07 a	43.95 ± 2.21 a	40.53 ±1.47 ab	3.42 ± 0.77 ab	904.77 ± 106.41 a	832.28 ± 85.00 a	72.49 ± 21.47 ab
	20–40	1.38 ± 0.10 a	40.87 ± 0.41 ab	37.81 ± 0.94 c	3.07 ± 0.56 ab	600.47 ± 52.14 a	556.3 ± 54.44 a	44.18 ± 5.82 b
	40–60	1.40 ± 0.05 a	40.55 ± 1.46 ab	37.27 ± 1.31 b	3.28 ± 0.48 ab	582.25 ± 35.27 b	535.43 ± 34.70 b	46.82 ± 6.25 ab
10	0–20	1.07 ± 0.01 b	41.08 ± 0.47 ab	40.40 ± 0.41 ab	0.68 ± 0.08 b	769.06 ± 5.99 a	756.20 ± 6.79 a	12.86 ± 1.21 b
	20–40	1.08 ± 0.02 a	40.87 ± 1.87 a	40.22 ± 1.78 ab	0.65 ± 0.10 b	758.88 ± 38.88 a	746.74 ± 37.44 a	12.13 ± 1.90 b
	40–60	1.31 ± 0.00 a	43.85 ± 0.23 a	43.17 ± 0.15 a	0.69 ± 0.23 c	668.89 ± 3.36 a	658.45 ± 4.21 a	10.44 ± 3.50 b
15	0–20	1.05 ± 0.07 a	43.85 ± 4.63 a	43.05 ± 4.63 a	0.80 ± 0.18 b	863.58 ± 119.72 a	848.52 ± 120.63 a	15.06 ± 2.87 b
	20–40	1.25 ± 0.06 ab	41.86 ± 0.49 a	40.45±0.09ab	1.41 ± 0.58 b	675.83 ± 26.64 ab	653.95 ± 33.96 a	21.88 ± 7.89 b
	40–60	1.30 ± 0.04 a	42.25±0.32a	40.82 ± 0.64 ab	1.44 ± 0.37 bc	655.08 ± 14.61 a	632.75 ± 17.66 a	22.33 ± 5.70 b
20	0–20	0.97 ± 0.05 a	38.20 ± 1.10 a	37.18 ± 0.92 ab	1.02 ± 0.30 b	796.45 ± 27.83 a	775.84 ± 32.68 a	20.61 ± 5.05 b
	20–40	1.13 ± 0.11 a	40.67 ± 0.96 ab	39.34 ± 0.62 bc	1.33 ± 0.67 bc	739.7 ± 87.77 a	716.92 ± 90.14 a	22.78 ± 9.87 b
	40–60	1.26 ± 0.07 ab	43.20 ± 0.58 a	41.90 ± 0.86 a	1.30 ± 0.35 b	691.58 ± 42.15 ab	670.49 ± 37.47 a	21.09 ± 6.88 b
42	0–20	1.10 ± 0.07 a	41.07 ± 1.91 a	34.55±0.45b	6.53 ± 2.16 a	757.70 ± 85.37 a	632.96 ± 38.59 a	124.74 ± 49.89 a
	20–40	1.24 ± 0.08 a	38.66 ± 2.08 b	33.79 ± 1.40 d	4.87 ± 1.00 a	634.75 ± 69.38 a	554.18 ± 53.77 a	80.57 ± 19.17 a
	40–60	1.30 ± 0.12 ab	37.78 ± 1.69 b	31.58 ± 2.37 c	6.20 ± 2.30 a	598.48 ± 82.55 b	494.25 ± 46.12 b	104.20 ± 47.02 a

Note: The data indicate mean ± standard error. Different lowercase letters indicated significant differences at the *p* < 0.05 level.

**Table 6:** Soil chemical content of *Michelia macclurei* plantations at different forest ages.

Stand Age (a)	Layer (cm)	Organic Matter (g·kg^−1^)	Total Nitrogen (g·kg^−1^)	Total Phosphorus (g·kg^−1^)	Total Potassium (g·kg^−1^)	Alkali-Hydrolyzable Nitrogen (mg·kg^−1^)	Available Phosphorus (mg·kg^−1^)	Available Potassium (mg·kg^−1^)	pH (H_2_O)
5	0–20	47.52 ± 7.98 a	1.76 ± 0.25 a	0.19 ± 0.02 ab	8.62 ± 0.42 b	185.56 ± 24.71 a	0.75 ± 0.20 a	38.73 ± 10.64 a	4.21 ± 0.02 ab
	20–40	17.84 ± 3.44 a	0.73 ± 0.13 b	0.14 ± 0.01 b	9.76 ± 0.74 b	75.94 ± 12.07 a	0.39 ± 0.03 a	25.43 ± 2.29 a	4.30 ± 0.02 bc
	40–60	12.65 ± 3.11 a	0.55 ± 0.04 b	0.14 ± 0.01c	10.25 ± 0.86 b	51.12 ± 6.16 a	0.35 ± 0.09 a	18.16±0.96a	4.33 ± 0.06 b
10	0–20	26.32 ± 7.14 b	0.96 ± 0.18 c	0.19±0.02 ab	6.65 ± 1.17 b	102.49 ± 14.35 b	0.75 ± 0.30 a	16.04 ± 1.56 b	4.15 ± 0.11 b
	20–40	18.24 ± 3.49 a	0.65 ± 0.08 b	0.18 ± 0.02 ab	7.12 ± 1.37 b	71.28 ± 5.98 a	0.42 ± 0.1 a	12.77 ± 1.35 b	4.23 ± 0.05 c
	40–60	10.42 ± 2.11 a	0.50 ± 0.04 b	0.17 ± 0.03 abc	7.45 ± 1.38 b	48.42 ± 8.24 a	0.22 ± 0.06 a	10.28 ± 0.87 bc	4.27 ± 0.04 b
15	0–20	39.03 ± 3.67 ab	1.44 ± 0.08 abc	0.23 ± 0.01 a	8.16 ± 0.20 b	132.72 ± 7.46 b	1.51 ± 1.05 a	14.14 ± 1.39 b	4.37 ± 0.04 ab
	20–40	13.31 ± 2.04 a	0.65 ± 0.06 b	0.20 ± 0.01 a	9.18 ± 0.41 b	57.27 ± 8.15 a	1.30 ± 0.75 a	8.55 ± 0.53 b	4.39 ± 0.04 ab
	40–60	11.25 ± 1.78 a	0.56 ± 0.04 b	0.20 ± 0.01 ab	9.54 ± 0.60 b	51.61 ± 6.85 a	0.19 ± 0.03 a	7.78 ± 0.42c	4.38 ± 0.04 ab
20	0–20	36.71 ± 2.33 ab	1.55 ± 0.06 ab	0.22 ± 0.02 a	17.43 ± 0.64 a	135.42 ± 7.99 b	0.85 ± 0.33 a	26.18 ± 2.12 ab	4.20±0.09ab
	20–40	23.56 ± 5.06 a	1.17 ± 0.16 a	0.21 ± 0.01 a	18.2 ± 0.81 a	93.64 ± 14.77 a	1.93 ± 1.66 a	21.32 ± 1.24 a	4.20 ± 0.03 c
	40–60	12.81 ± 2.17 a	0.86 ± 0.09 a	0.22 ± 0.01 a	20.27 ± 1.06 a	60.71 ± 8.30 a	1.15 ± 0.74 a	17.23 ± 0.96 ab	4.26 ± 0.04 b
42	0–20	31.83 ± 2.10 ab	1.08 ± 0.06 bc	0.16 ± 0.02 b	7.37 ± 3.99 a	105.44 ± 6.65 b	0.75 ± 0.28 a	22.33 ± 1.63 ab	4.39 ± 0.03 a
	20–40	21.63 ± 4.49 a	0.83 ± 0.14 ab	0.15 ± 0.02 b	18.97 ± 4.89 a	79.14 ± 15.45 a	0.45 ± 0.12 a	22.51 ± 2.78 a	4.44 ± 0.02 a
	40–60	11.54 ± 2.66 a	0.54 ± 0.09 b	0.15 ± 0.02 bc	19.61 ± 5.38 a	55.79 ± 12.08 a	0.42 ± 0.10 a	19.21 ± 4.90 a	4.51 ± 0.02 a

Note: The data indicate mean ± standard error. Different lowercase letters indicated significant differences at the *p* < 0.05 level.

## Data Availability

Data are available upon request.
